# Identification of hub genes and candidate drugs in hepatocellular carcinoma by integrated bioinformatics analysis

**DOI:** 10.1097/MD.0000000000027117

**Published:** 2021-10-01

**Authors:** Xiaolong Chen, Zhixiong Xia, Yafeng Wan, Ping Huang

**Affiliations:** a National Key Clinical Department, Department of Hepatobiliary Surgery, The First Affiliated Hospital of Chongqing Medical University, Chongqing, China; b Department of Pathology, The Center Hospital of Wuhan, Hubei, China; c Department of Hepatobiliary Surgery, Daping Hospital, Army Medical University, Chongqing, China.

**Keywords:** bioinformatics analysis, differently expressed genes, drug, hepatocellular carcinoma, hub genes

## Abstract

**Background:**

Hepatocellular carcinoma (HCC) is the third cancer-related cause of death in the world. Until now, the involved mechanisms during the development of HCC are largely unknown. This study aims to explore the driven genes and potential drugs in HCC.

**Methods:**

Three mRNA expression datasets were used to analyze the differentially expressed genes (DEGs) in HCC. The bioinformatics approaches include identification of DEGs and hub genes, Gene Ontology terms analysis and Kyoto encyclopedia of genes and genomes enrichment analysis, construction of protein–protein interaction network. The expression levels of hub genes were validated based on The Cancer Genome Atlas, Gene Expression Profiling Interactive Analysis, and the Human Protein Atlas. Moreover, overall survival and disease-free survival analysis of HCC patients were further conducted by Kaplan–Meier plotter and Gene Expression Profiling Interactive Analysis. DGIdb database was performed to search the candidate drugs for HCC.

**Results:**

A total of 197 DEGs were identified. The protein–protein interaction network was constructed using Search Tool for the Retrieval of Interacting Genes software, 10 genes were selected by Cytoscape plugin cytoHubba and served as hub genes. These 10 genes were all closely related to the survival of HCC patients. DGIdb database predicted 29 small molecules as the possible drugs for treating HCC.

**Conclusion:**

Our study provides some new insights into HCC pathogenesis and treatments. The candidate drugs may improve the efficiency of HCC therapy in the future.

## 1 Introduction

Hepatocellular carcinoma (HCC) is one of the major health problems worldwide.^[1,2]^ It affects more than half a million people worldwide every year, with about a 30% 5-year survival rate.^[3,4]^ Although a variety of therapies have been used to treat HCC in the past few decades, the treatment effect is still unsatisfactory due to postoperative recurrence and drug resistance. Increasing evidence has shown that the molecular pathogenesis of HCC may be closely associated with living environment and genetic factors, such as P53 inactivation, several oncogene activation, and gene mutation.^[5,6]^ However, the precise mechanisms underlying HCC development and progression remain unclear.

Recently, the rapid development of high-throughput RNA microarray analysis has allowed us to better understand the underlying mechanisms and general genetic alterations involved in HCC occurrence and metastasis. RNA microarrays have been extensively applied to explore HCC carcinogenesis through gene expression profiles and the identification of altered genes.^[7–9]^ Meanwhile, many large public databases such as The Cancer Genome Atlas (TCGA), and Gene Expression Omnibus (GEO) can be performed to screen the differentially expressed genes (DEGs) related to the initiation and progression of HCC from microarray data.

Most HCC patients have a relatively long latent period, therefore many HCC patients are in the intermediate or advanced stage when first diagnosed, in which case radical surgery is no longer desirable.^[10]^ However, many chemotherapies are often with unsatisfactory curative effects and some severe side effects. For example, sorafenib shows a 3-month median survival benefit but is related to 2 grade 3 drug-related adverse events namely diarrhea and hand-foot skin reaction.^[11]^ At present, the disease-free survival (DFS) and overall survival (OS) of HCC patients remained relatively short, highlighting the importance of developing new drugs.

In the study, 3 mRNA expression profiles were downloaded (GSE121248,^[12]^ GSE64041,^[13]^ and GSE62232^[14]^) from the GEO database to identify the genes correlated to HCC progression and prognosis. Integrated analysis included identifying DEGs using the GEO2R tool, overlapping 3 datasets using a Venn diagram tool, GO terms analysis, KEGG biological pathway enrichment analysis, protein–protein interaction (PPI) network construction, hub genes identification and verification, construction of hub genes interaction network, survival analysis of these screened hub genes, and exploration of candidate small molecular drugs for HCC.

## 2 Materials and methods

### 2.1 Data collection

HCC and adjacent normal tissue gene expression profiles of GSE 121248, GSE64041, and GSE62232 were downloaded from the GEO database (http://www.ncbi.nlm.nih.gov/geo/).^[15]^ The microarray data of GSE121248 was based on GPL571 Platforms (Affymetrix Human Genome U133 Plus 2.0 Array) and included 70 HCC tissues and 37 normal tissues (Submission date: October 15, 2018). The GSE64041 data was based on GPL6244 Platforms (Affymetrix Human Gene 1.0 ST Array) and included 60 biopsy pairs from HCC patients, 5 normal liver biopsies (Submission date: December 10, 2014). The data of GSE62232 was based on GPL571 Platforms (Affymetrix Human Genome U133 Plus 2.0 Array) and included 81 HCC cancer tissues and 10 normal liver tissues (Submission date: October 9, 2014). The above datasets meet the following criteria: they used tissue samples from human HCC tissues and adjacent or non-tumor liver tissues; each dataset involved more than 90 samples.

### 2.2 DEGs identification

GEO2R (https://www.ncbi.nlm.nih.gov/geo/geo2r/) was used to screen the DEGs in HCC tumor tissues and non-tumor liver tissues.^[16]^ Adjusted *P* values (adj. *P*) < .05 and |logFC| > 1 were set as the cutoff criterion to select DEGs for every dataset microarray, respectively.^[17]^ Then, the overlapping DEGs among these 3 datasets were identified by the Venn diagram tool (https://bioin fogp.cnb.csic.es/tools/venny/). Visual hierarchical cluster analysis was also performed to display the volcano plot of DEGs.

### 2.3 GO and KEGG pathway enrichment analysis

To explore the functions of these DEGs, the DAVID database (https://david.ncifcrf.gov/) was used to perform GO term analysis at first.^[18]^ Then we submitted these DEGs, including 54 upregulated genes and 143 downregulated genes, into the Enrichr database to perform KEGG pathway enrichment analysis. GO term consisted of the following 3 parts: biological process, cellular component, and molecular function. Adj. *P* < .05 was regarded as statistically significant.

### 2.4 Construction of PPI network and screening of hub genes

PPI network is the network of protein complexes due to their biochemical or electrostatic forces. The Search Tool for the Retrieval of Interacting Genes (STRING) (https://string-db.org/cgi/input .pl/) is a database constructed for analyzing the functional proteins association network.^[19]^ The screened DEGs had been submitted to the STRING database, and all PPI pairs with a combined score of >0.4 were extracted. The degree of all nodes was calculated by Cytoscape (v3.6.1) plugin cytoHubba.^[20]^ In the study, these genes with the top 10 highest degree values were regarded as hub genes.

### 2.5 Validation of hub genes

To validate the mRNA expression level of the hub genes in HCC, the Gene Expression Profiling Interactive Analysis (GEPIA) database was used to show the difference in the mRNA expression level of each hub gene between the liver hepatocellular carcinoma (LIHC) and non-cancerous liver samples.^[21]^ Afterward, the protein expression levels of the hub genes in normal and HCC tissues were visualized through The Human Protein Atlas (HPA) database that contains immunohistochemistry-based expression data for about 20 common types of cancers.^[22]^

### 2.6 Genetic alterations of hub genes

The LIHC dataset (TCGA, PanCancer Atlas) including the data of 348 samples was selected to analyze the genetic alterations of hub genes using the cBioPortal database. This database allows for visualization, analysis, and downloading a lot of cancer genomic datasets.^[23]^ These genomic alterations included gene mutations, copy number variations, deep deletion, mRNA expression *z*-scores (RNA Seq V2 RSEM) with a *z*-score threshold of ±2.0, and protein expression *z*-scores. According to the online instructions of cBioPortal, the analysis on DFS and OS was also carried out.

### 2.7 Survival analysis for hub genes

Kaplan–Meier plotter is extensively applied to explore the roles of more than 54,000 genes in OS based on 13,316 tumor samples from GEO, the European Genome-phenome Archive, and TCGA datasets including 364 patients with liver cancer. The relation between OS and hub genes expressed in patients with liver cancer was determined by the Kaplan–Meier survival analysis.^[24]^ Moreover, the relation between DFS and these genes expressed in LIHC patients was explored through the online tool GEPIA database. The lower and upper 50% of gene expression were set as the standard for analysis. In the present study, HCC patients were divided into 2 groups based on the median expression values of the hub genes. Log-rank *P* < .01 was regarded as statistically significant.

### 2.8 Drug-hub gene interaction

The screened hub genes were also regarded as promising targets for searching drugs through the DGIdb database (http://dgidb.genome.wustl.edu/).^[25]^ This database has drug–gene interaction data from 30 disparate sources such as ChEMBL, DrugBank, Ensembl, NCBI Entrez, PharmGKB, and literature in NCBI PubMed. Drugs supported by no less than 2 databases or PubMed references were validated as the candidate drugs. The final list only contained the drugs that have been approved by the Food and Drug Administration. Additionally, the identified target gene network was constructed through the STITCH database (http://stitch.embl.de/), a software that also incorporated drug–gene relationships.^[26,27]^

## 3 Results

### 3.1 Identification of DEGs

**Figure 1:**
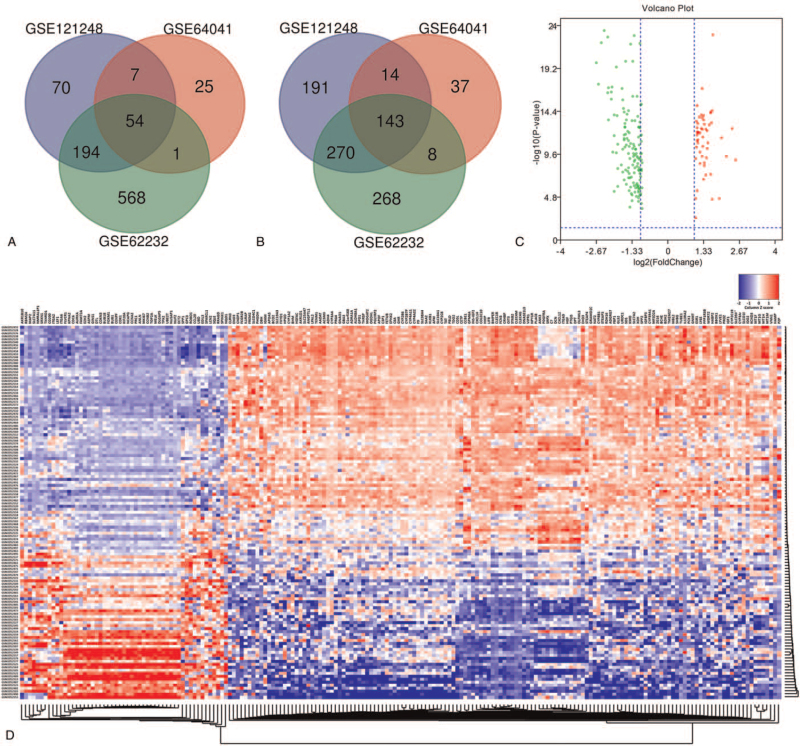
Identification of common DEGs from GSE121248, GSE64041, and GSE62232 datasets. Venn diagram of (A) upregulated and (B) downregulated DEGs based on the 3 GEO datasets. (C) Volcano plot of the 197 DEGs. Red, upregulation; green, downregulation. The intersecting areas represent the commonly altered DEGs. (D) The heatmap of 197 DEGs using data profile GSE64041 as a reference. The *t* test was used to analyze DEGs, with the cutoff criteria of |logFC| > 1.0 and adj. *P* < .05. DEGs = differentially expressed genes, GEO = Gene Expression Omnibus, logFC = log-fold change.

According to GSE121248 dataset analysis, 943 DEGs were successfully identified, including 325 upregulated and 618 downregulated genes. For GSE64041 dataset, 289 DEGs were observed, including 87 upregulated and 202 downregulated genes. For GSE62232 dataset, 1355 DEGs were identified, involving 817 upregulated and 538 downregulated genes. Venn analysis was performed to examine the intersection among the 3 DEGs profiles. Then, 197 DEGs were identified from the 3 profile datasets (Table 1). Obviously, 54 DEGs were significantly upregulated (Fig. 1A), while 143 DEGs were markedly downregulated (Fig. 1B) in HCC tissues. These 197 DEGs were plotted in Fig. 1C, where the red and green dots represented the upregulated and downregulated DEGs, respectively. In addition, the mRNA expression level of these 197 DEGs was visualized in the form of a heatmap using data profile GSE64041 (Fig. 1D).

**Table 1:** The common DEGs of 3 gene expression profiles.

DEGs	Gene symbol
Upregulated (54)	SPINK1; TPX2; EDIL3; ASPM; FLVCR1; AKR1B10; GINS1; SRXN1; KPNA2; ANLN; NQO1; FOXM1; EZH2; CCNB2; RBM24; PRC1; CDK1; TOP2A; TXNRD1; SPARCL1; CDC6; FAM72A; MAP2; AURKA; BUB1B; DLGAP5; NMRAL1P1; LEF1; MKI67; CAP2; DTL; GPC3; CCL20; ROBO1; SPP1; SQLE; KIF20A; UBD; RRAGD; CD200; ITGA6; LCN2; MELK; SLC7A11; ITGA2; CCNA2; CDKN3; BUB1; NUF2; NCAPG; UBE2T; CENPF; NUSAP1; ECT2
Downregulated (143)	TUBE1; BBOX1; XDH; SDS; CXCL14; IGF1; DPT; CYP39A1; SLC25A47; PROZ; C8A; ZG16; MBL2; SLC10A1; SLCO1B3; PRG4; CYP1A2; UROC1; FCGR2B; F9; BCO2; ACSM3; CYP2C19; C3P1; LPA; CD5L; GHR; CLEC1B; TAT; LIFR; BHMT; COLEC10; VNN1; LYVE1; STEAP3; SHBG; DNASE1L3; ALDH8A1; NAT2; C7; BCHE; SAA2-SAA4; AKR1D1; CXCL12; GNMT; C1orf168; GPD1; CRHBP; EHD3; WDR72; IDO2; BDH2; CYP3A43; SLC38A4; DBH; FBP1; ADH4; OIT3; MT1M; SLC39A5; CETP; SRD5A2; ADRA1A; PBLD; SRPX; CYP4A22; KLKB1; GNAO1; ENO3; MT1G; SLC19A3; PGLYRP2; TENM1; INS-IGF2; CYP2C8; STEAP4; IL13RA2; SPP2; IGHM; MT1F; FETUB; MFSD2A; HHIP; APOA5; CYP2B7P; KCND3; PPP1R3B; LY6E; ITGA9; OLFML3; CNDP1; FCN3; GBA3; PDGFRA; CLEC4G; PHGDH; CYP2B6; CCBE1; FXYD1; PCK1; KMO; ANK3; CLRN3; MT1H; CLEC4M; NPY1R; ESR1; TDO2; VIPR1; IGFBP3; PLAC8; HAMP; DCN; IL1RAP; RDH16; CYP8B1; TMEM27; AFM; HPGD; LPAL2; THRSP; CYP4A11; STAB2; HGFAC; ADGRG7; OGDHL; PZP; SLCO4C1; FREM2; BMPER; AADAT; GPM6A; HGF; MOGAT2; CYP3A4; EPHX2; GLS2; HABP2; APOF; ANGPTL1; PTGIS; GRAMD1C; SLC7A2

DEGs =  differentially expressed genes.

### 3.2 Functional enrichment analysis of DEGs

GO annotation and KEGG pathways enrichment analysis were conducted through the DAVID database and Enrichr database, respectively. The top 10 enriched GO term and KEGG pathways were showed in Table 2. As shown in Table 2, GO biological process analysis revealed that these 197 DEGs were significantly enriched in the oxidation-reduction process, organic acid metabolic process, carboxylic acid metabolic process, and oxoacid metabolic process. The top 4 significantly enriched cellular components terms included extracellular space, extracellular region part, extracellular region, and pronucleus. For GO molecular function analysis, the top 4 significantly enriched terms were monooxygenase activity, oxidoreductase activity, heme binding, and iron ion binding. Additionally, the top 4 markedly enriched pathways for these 197 DEGs were metabolic pathways, tryptophan metabolism, chemical carcinogenesis, and caffeine metabolism.

**Table 2:** Functional and pathway enrichment analysis of the common DEGs.

Category	Term	Count	*P* value
GOTERM_BP_FAT	GO:0055114∼oxidation-reduction process	41	5.26E-13
GOTERM_BP_FAT	GO:0006082∼organic acid metabolic process	38	4.31E-12
GOTERM_BP_FAT	GO:0019752∼carboxylic acid metabolic process	36	6.26E-12
GOTERM_BP_FAT	GO:0043436∼oxoacid metabolic process	36	7.41E-12
GOTERM_BP_FAT	GO:0032787∼monocarboxylic acid metabolic process	29	2.30E-11
GOTERM_BP_FAT	GO:1901565∼organonitrogen compound catabolic process	19	1.22E-07
GOTERM_BP_FAT	GO:0008202∼steroid metabolic process	16	4.69E-07
GOTERM_BP_FAT	GO:0019373∼epoxygenase P450 pathway	6	1.22E-06
GOTERM_BP_FAT	GO:0040007∼growth	29	1.47E-06
GOTERM_BP_FAT	GO:0017144∼drug metabolic process	7	2.15E-06
GOTERM_CC_FAT	GO:0005615∼extracellular space	44	1.16E-07
GOTERM_CC_FAT	GO:0044421∼extracellular region part	80	2.66E-06
GOTERM_CC_FAT	GO:0005576∼extracellular region	90	4.40E-06
GOTERM_CC_FAT	GO:0045120∼pronucleus	4	8.52E-04
GOTERM_CC_FAT	GO:1903561∼extracellular vesicle	55	0.001147
GOTERM_CC_FAT	GO:0043230∼extracellular organelle	55	0.001157
GOTERM_CC_FAT	GO:1990777∼lipoprotein particle	5	0.001538
GOTERM_CC_FAT	GO:0034358∼plasma lipoprotein particle	5	0.001538
GOTERM_CC_FAT	GO:0005887∼integral component of plasma membrane	36	0.001686
GOTERM_CC_FAT	GO:0070062∼extracellular exosome	54	0.001780
GOTERM_MF_FAT	GO:0004497∼monooxygenase activity	15	1.39E-11
GOTERM_MF_FAT	GO:0016705∼oxidoreductase activity	16	9.28E-10
GOTERM_MF_FAT	GO:0020037∼heme binding	14	7.03E-09
GOTERM_MF_FAT	GO:0005506∼iron ion binding	15	1.06E-08
GOTERM_MF_FAT	GO:0046906∼tetrapyrrole binding	14	1.52E-08
GOTERM_MF_FAT	GO:0019825∼oxygen binding	8	1.68E-06
GOTERM_MF_FAT	GO:0048037∼cofactor binding	14	1.88E-05
GOTERM_MF_FAT	GO:0016614∼oxidoreductase activity	10	3.18E-05
GOTERM_MF_FAT	GO:0008395∼steroid hydroxylase activity	6	3.67E-05
GOTERM_MF_FAT	GO:0016709∼oxidoreductase activity	6	1.35E-04
KEGG_PATHWAY	hsa01100:Metabolic pathways	34	1.49E-04
KEGG_PATHWAY	hsa00380:Tryptophan metabolism	6	2.58E-04
KEGG_PATHWAY	hsa05204:Chemical carcinogenesis	7	0.001024
KEGG_PATHWAY	hsa00232:Caffeine metabolism	3	0.002033
KEGG_PATHWAY	hsa00830:Retinol metabolism	6	0.002271
KEGG_PATHWAY	hsa00982:Drug metabolism – cytochrome P450	6	0.002967
KEGG_PATHWAY	hsa00591:Linoleic acid metabolism	4	0.008281
KEGG_PATHWAY	hsa00590:Arachidonic acid metabolism	5	0.011650
KEGG_PATHWAY	hsa01130:Biosynthesis of antibiotics	9	0.011704
KEGG_PATHWAY	hsa04115:p53 signaling pathway	5	0.016021

DEGs =  differentially expressed genes, GO = Gene Ontology, KEGG = Kyoto encyclopedia of genes and genomes.

### 3.3 PPI network construction and hub genes identification

The STRING database was performed to determine the PPI network among the 197 DEGs. The PPI network including 197 nodes (genes) and 968 edges (interactions) was constructed through the STRING database (see Fig. S1, Supplemental Digital Content, which shows the PPI network constructed). The PPI enrichment *P* value <1.0 × 10^−16^. Ten genes with the highest degree scores were regarded as the hub genes by applying the Cytoscape (v3.6.1) plugin cytoHubba. The results revealed that forkhead box M1 (FOXM1) was the hub gene with the highest connectivity degree, followed by aurora kinase A (AURKA), cyclin A2 (CCNA2), cyclin-dependent kinase inhibitor 3 (CCKN3), marker of proliferation Ki-67 (MKI67), enhancer of zeste 2 polycomb repressive complex 2 subunit (EZH2), cell division cycle 6 (CDC6), cyclin-dependent kinase 1 (CDK1), cyclin B1 (CCNB1), Topoisomerase (DNA) II alpha (TOP2A) (Table 3).

**Table 3:** Top 10 hub genes with higher degree of connectivity.

Gene symbol	Gene description	Degree
FOXM1	Forkhead box M1	36
AURKA	Aurora kinase A	34
CCNA2	Cyclin A2	34
CDKN3	Cyclin-dependent kinase inhibitor 3	34
MKI67	Marker of proliferation Ki-67	34
EZH2	Enhancer of zeste 2 polycomb repressive complex 2 subunit	33
CDC6	Cell division cycle 6	33
CDK1	Cyclin-dependent kinase 1	33
CCNB1	Cyclin B1	33
TOP2A	Topoisomerase (DNA) II alpha	33

**Figure 2:**
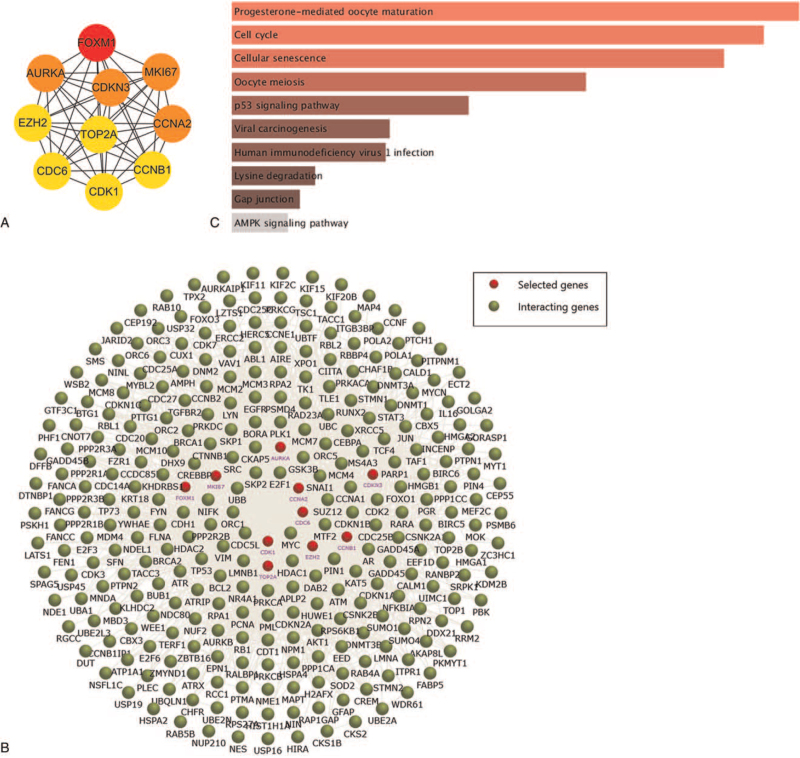
Interaction network and KEGG analysis of the hub genes. (A) The top 10 hub genes in the PPI network were screened by Cytoscape (v3.6.1) plugin cytoHubba. The 10 hub genes are displayed from red (high degree value) to yellow (low degree value). (B) The PPI network of the 10 hub genes and their related genes, created by the FunRich software. (C) KEGG pathway enrichment analysis of the 10 hub genes. KEGG = Kyoto encyclopedia of genes and genomes, PPI = protein–protein interaction, STRING = search tool for the retrieval of interacting genes.

Using cytoHubba software, the PPI network of the screened 10 hub genes was constructed, which had a strong interaction among each other (Fig. 2A). The interaction network of 10 hub genes and their related genes was also identified by the FunRich software (Fig. 2B).^[28]^ The hub genes and their related genes could be enriched in many biological pathways through the enrichment functions of the FunRich tool. KEGG analysis established that markedly enriched pathways for the hub genes included progesterone mediated oocyte maturation, cell cycle, cellular senescence, oocyte meiosis, p53 signaling pathway, viral carcinogenesis, lysine degradation, and gap junction (Fig. 2C).

### 3.4 Validation of hub genes in HCC

**Figure 3:**
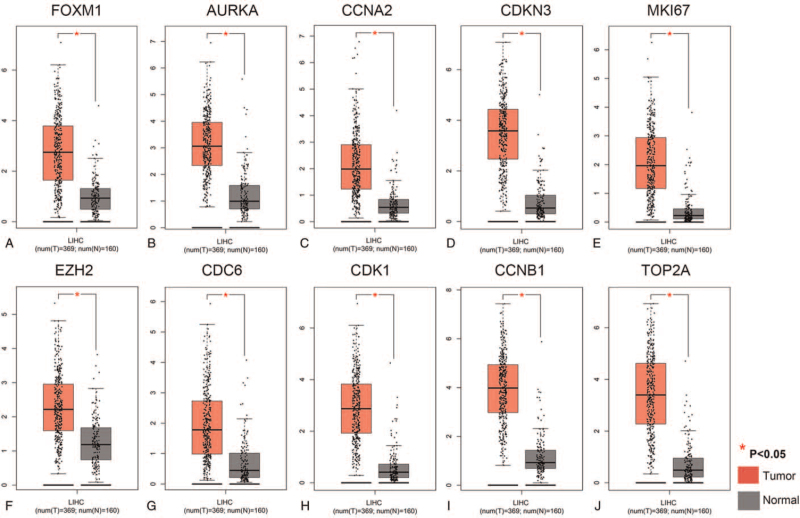
Validation of the mRNA expression levels of (A) FOXM1, (B) AURKA, (C) CCNA2, (D) CCKN3, (E) MKI67, (F) EZH2, (G) CDC6, (H) CDK1, (I) CCNB1, and (J) TOP2A in LIHC tissues and normal liver tissues using GEPIA database. These 10 box plots are based on 369 LIHC samples (marked in red) and 160 normal samples (marked in gray). ^∗^*P* < .01 was considered statistically significant. LIHC = liver hepatocellular carcinoma.

First, a differential analysis on the mRNA expression levels of FOXM1, AURKA, CCNA2, CCKN3, MKI67, EZH2, CDK1, CCNB1, and TOP2A, between HCC and non-tumor liver tissues was conducted through the GEPIA database. As shown in Figure 3, the mRNA expression levels of (Fig. 3A) FOXM1, (Fig. 3B) AURKA, (Fig. 3C) CCNA2, (Fig. 3D) CCKN3, (Fig. 3E) MKI67, (Fig. 3F) EZH2, (Fig. 3G) CDC6, (Fig. 3H) CDK1, (Fig. 3I) CCNB1, and (Fig. 3J) TOP2A were significantly upregulated in HCC tissues (*P* < .01) compared to those in normal liver tissues. These findings were consistent with the obtained GEO microarray data.

Moreover, the protein expression levels of these hub genes in HCC were validated through the HPA database. Obviously, the protein expression levels of FOXM1, AURKA, CCNA2, MKI67, EZH2, CDC6, CDK1, CCNB1, and TOP2A were not observed or low in normal liver tissues, but medium or high expression levels of these hub genes were detected in HCC tissues (see Fig. S2, Supplemental Digital Content, which demonstrates protein expression levels of these hub genes in HCC). Unfortunately, the protein expression levels of CDKN3 were not explored because of pending cancer tissue analysis in the HPA database. In brief, these present results showed that mRNA and protein expression levels of these hub genes were overexpressed in HCC tissues.

### 3.5 Survival analysis of the hub genes in HCC

**Figure 4:**
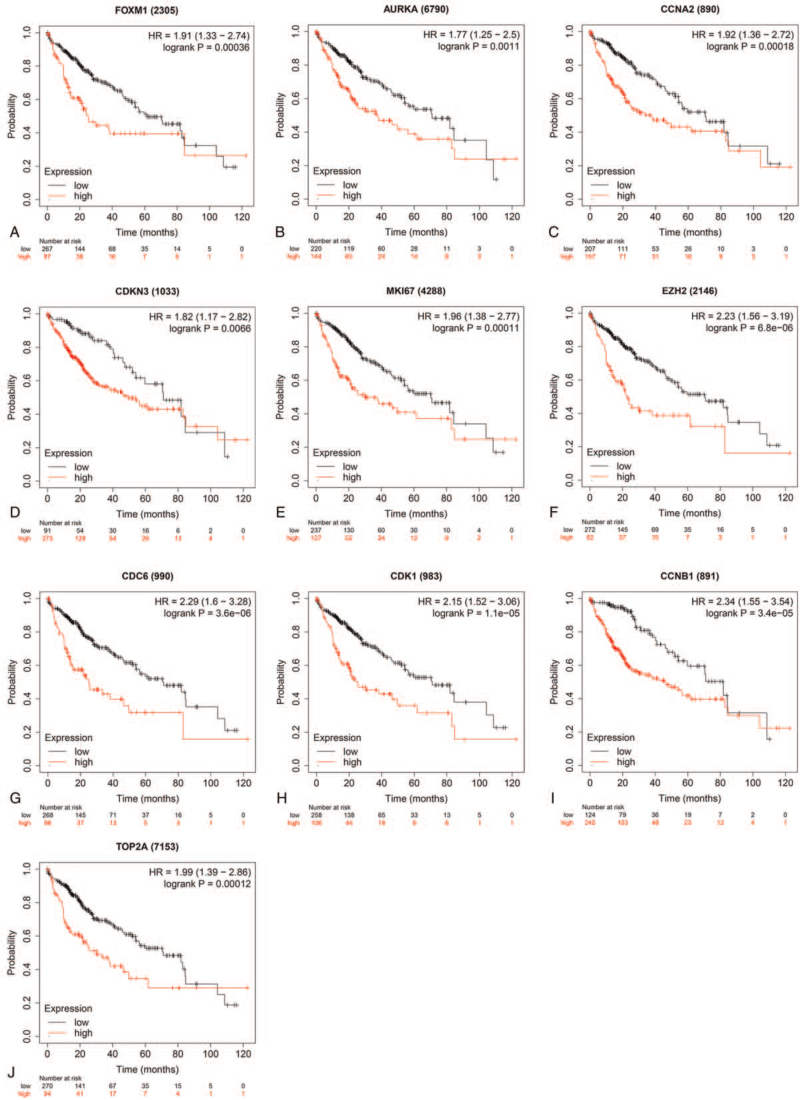
OS of the 10 hub genes overexpressed in patients with liver cancer was analyzed by Kaplan–Meier plotter. FOXM1, log-rank *P* = .00036; AURKA, log-rank *P* = .0011; CCNA2, log-rank *P* = .00018; CDKN3, log-rank *P* = .0066; MKI67, log-rank *P* = .00011; EZH2, log-rank P = 6.8e-06; CDC6, log-rank P = 3.6e-06; CDK1, log-rank P = 1.1e-05; CCNB1, log-rank P = 3.4E-05; and TOP2A, log-rank *P* = .00012. Data are presented as Log-rank P and the hazard ratio with a 95% confidence interval. Log-rank *P* < .01 was regarded as statistically significant. OS = overall survival.

To further explore the relationship between the 10 hub genes and HCC, OS, and DFS analysis of the 10 hub genes were performed by Kaplan–Meier plotter, and the GEPIA database. As showed in Figure 4, high expression levels of FOXM1, AURKA, CCNA2, CDKN3, MKI67, EZH2, CDC6, CDK1, CCNB1, and TOP2A in LIHC patients were related to poor OS. The unfavorable DFS was also significantly shown in LIHC patients with high expression levels of the 10 hub genes (see Fig. S3, Supplemental Digital Content, which illustrates DFS of LIHC patients overexpressed the 10 hub genes).

### 3.6 Drug-hub gene interaction

Using the DGIdb database to explore drug-gene interactions of the 10 hub genes, 29 drugs for possibly treating HCC were matched and determined (Table 4). Promising targeted genes of these drugs include AURKB, EZH2, and TOP2A. The final list only included these drugs which were approved by Food and Drug Administration, and several drugs have been tested in clinical trials. Paclitaxel was considered a potential drug for cancer therapy due to its inhibition of AURKA and TOP2A. Etoposide, an inhibitor of TOP2A, could inhibit the development of cancer by inducing DNA damage. Using the STITCH database, we constructed downstream networks of AURKA, EZH2, and TOP2A to investigate the additional effects caused by inhibitors of these genes. Our models showed that AURKA inhibition might have a possible influence on TPX2, microtubule nucleation factor (TPX2), cell division cycle 20 (CDC20), tumor protein p53 (TP53), cell division cycle 25B (CDC25B), baculoviral IAP repeat-containing 5 (BIRC5); EZH2 inhibition might have possible influence on histone deacetylase 1 (HDAC1), BMI1 proto-oncogene, polycomb ring finger (BMI1), YY1 transcription factor (YY1), DNA methyltransferase 3 alpha (DNMT3A), DNA methyltransferase 3 beta (DNMT3B), DNA methyltransferase 1 (DNMT1), RB binding protein 4 (RBBP4), embryonic ectoderm development (EED); TOP2A inhibition might have a possible influence on DNA topoisomerase I (TOP1), DNA topoisomerase II beta (TOP2B), ubiquitin C (UBC), proliferating cell nuclear antigen (PCNA), small ubiquitin-like modifier 1 (SUMO1), and SUMO2 (see Figs. S4–S6, Supplemental Digital Content, which shows downstream networks of AURKA, EZH2, and TOP2A respectively). So far, few inhibitors of AURKA, EZH2, and TOP2A have been tested for HCC therapy. Some of these drugs were even not regarded as anti-cancer drugs (such as levofloxacin and dexrazoxane). These data could provide new insights for targeted therapy in HCC patients.

**Table 4:** Candidate drugs targeting hub genes.

Number	Gene	Drug	Interaction types	Score^∗^	PubMed ID
1	AURKA	PACLITAXEL	–	2	12559175
2	AURKA	TAMOXIFEN	–	2	24166501
3	AURKA	FLUOROURACIL	–	2	25924824
4	CCNA2	ETHINYL ESTRADIOL	–	2	9806355
5	EZH2	DOXORUBICIN	–	2	25605023
6	EZH2	VORINOSTAT	–	2	25605023
7	EZH2	DABRAFENIB	–	2	27135738
8	EZH2	SULFINPYRAZONE	–	2	28135237
9	TOP2A	TENIPOSIDE	Inhibitor	12	8702194;16271071;17361331;17514873;11752352;16480143;9426516
10	TOP2A	ETOPOSIDE	Inhibitor	12	8823806;9485461;8870683;9494516;9426516
11	TOP2A	VINCRISTINE	–	10	9494516
12	TOP2A	DOXORUBICIN	Inhibitor	4	–
13	TOP2A	NORFLOXACIN	Inhibitor	2	11752352
14	TOP2A	VALRUBICIN	Inhibitor	6	11752352;16019763
15	TOP2A	LEVOFLOXACIN	Inhibitor	2	11752352
16	TOP2A	ENOXACIN	Inhibitor	4	18471102;11752352;10089819
17	TOP2A	DAUNORUBICIN	–	3	9494516
18	TOP2A	OFLOXACIN	Inhibitor	2	2847647
19	TOP2A	PEFLOXACIN	Inhibitor	2	11752352
20	TOP2A	AMSACRINE	Inhibitor	12	1322791;8823806;10691026;8519659;8632768; 11006484;11716434;11752352;11473732;1311390
21	TOP2A	PODOFILOX	Inhibitor	9	16061385;1334447;10783066;11752352;1845848;1331331
22	TOP2A	DEXRAZOXANE	–	2	12911317
23	TOP2A	MITOXANTRONE	Inhibitor	13	10451375;11004693;18687447;11752352; 9631585;9494516;11278845;9426516
24	TOP2A	LOMEFLOXACIN	Inhibitor	2	11752352
25	TOP2A	EPIRUBICIN	Inhibitor	6	14728934;16234514;17639997
26	TOP2A	DACTINOMYCIN	–	2	9494516
27	TOP2A	FINAFLOXACIN	Inhibitor	2	25808831
28	TOP2A	IDARUBICIN	–	2	–
29	TOP2A	HYDROQUINONE	–	2	15833037

∗ The score is the combined number of database sources and PubMed references supporting a given interaction.

## 4 Discussion

In the present study, bioinformatics analysis was performed to identify the potential key genes and biological pathways in HCC. Through comparing the 3 DEGs profiles of HCC obtained from the GEO database, 54 upregulated DEGs and 143 downregulated DEGs were identified respectively (Fig. 1). Based on the degree of connectivity in the PPI network, the 10 hub genes were screened and ranked, including FOXM1, AURKA, CCNA2, CDKN3, MKI67, EZH2, CDC6, CDK1, CCNB1, and TOP2A. These 10 hub genes were functioned as a group and may play a key role in the incidence and prognosis of HCC (Fig. 2A). HCC cases with high expression of the hub genes exhibited significantly worse OS and DFS compared to those with low expression of the hub genes (Fig. 4, Fig. S3). Additionally, 29 identified drugs provided new insights into targeted therapies of HCC (Table 4).

Retinol metabolism, arachidonic acid metabolism, tryptophan metabolism, and caffeine metabolism were most markedly enriched for HCC through KEGG pathway enrichment analysis for 197 DGEs. Metabolic alterations clearly characterize HCC tumors.^[29,30]^ Currently, the rapid development of metabolomics that allows metabolite analysis in biological fluids is very useful for discovering new biomarkers. Lots of new metabolites have been identified by metabolomics approaches, and some of them could be used as biomarkers in HCC.^[31]^

According to the degree of connectivity, the top 10 genes in the PPI network were regarded as hub genes and they were validated in the GEPIA database, UCSC Xena browser, and HPA database. Many studies reveal that the fork-head box transcription factor FOXM1 is essential for HCC development.^[32–34]^ Over-expression of FOXM1 has been exhibited to be strong relative to poor prognosis and progression of HCC.^[35,36]^ Hepatic progenitor cells of HCC have been identified in the chemical carcinogenesis model, they express cell surface markers CD44 and EpCAM.^[32,37]^ Interestingly, deletion of FOXM1 causes the disappearance of those cells in the tumor nodules, showing that FOXM1 is critical for the CD44 and EpCAM positive HCC cells.^[32]^ The hepatic cancer stem cells in human HCC lines also depend on FOXM1, because deletion of FOXM1 will lead to loss of these cancer stem cells.^[32]^ FOXM1 is a critical downstream factor of many cancer signaling pathways, such as Wnt/β-catenin signaling.^[38]^ Moreover, FOXM1 stimulates the expression of some multifunctional genes, like c-Myc, Oct4, Sox2, and Nanog.^[39,40]^

AURKA is a mitotic serine/threonine kinase that regulates cell mitosis, cell division, and cell cycle progression.^[41]^ AURKA overexpression has been observed in HCC.^[42]^ And AURKA overexpression has been closely relative to the aggressive tumor characteristics,^[43]^ poor prognosis,^[44]^ and drug resistance^[45]^ of HCC. AURKA was regulated by c-Myc which contributes to cancer progression in HCC.^[46]^ Alisertib, an inhibitor of AURKA, could inhibit cell viability and induce apoptosis in HCC cells.^[47]^ Wang et al showed genetic variations of AURKA may be a reliable biomarker for the development of HCC.^[48]^ Our study also indicated that increased expression levels of AURKA were relative to the unfavorable OS and DFS in HCC patients.

CCNA2^[49]^ and CCNB1^[50]^ are 2 members of the cyclin family, which regulate cell proliferation and apoptosis, and have been closely related to cancer progress and patients’ survival. CCNA2^[51]^ and CCNB1^[52,53]^ have been identified in various types of tumors. CCNA2 was overexpressed in human HCC tissues.^[54]^ Moreover, it was reported that CCNA2 was relative to a decrease in OS for HCC patients, based on the survival and expression data from TCGA.^[55]^ Liu et al revealed that CCNB1 was highly expressed in HCC tissues compared with normal liver tissues.^[56]^ In addition, the overexpression of CCNB1 was correlated to poor OS and DFS in HCC patients by bioinformatics analysis.^[57]^ Our study also revealed that HCC patients with a high expression level of CCNA2 or CCNA2 exhibited worse OS and DFS compared to those with a low expression level.

CDKN3 gene is involved in cell mitosis by modulating CDK1/CDK2 dephosphorylation, and its overexpression correlates with unfavorable survival in several cancers.^[58]^ For HCC, CDKN3 not only promotes cell proliferation but also correlates with tumor pathological grade negatively.^[59]^ CDK1, a member of the Ser/Thr protein kinase family, plays an essential role in the control of the eukaryotic cell cycle by modulating the centrosome cycle. CDK1 has been extensively investigated in ovarian cancer and colorectal cancer.^[60,61]^ However, little is known about the role of CDK1 in HCC carcinogenesis. A recent study has found that metformin can significantly inhibit the proliferation of HCC cells and effectively reduce the expression of CDK1.^[62]^ In the present study, the high expression of CDK1 is associated with unfavorable OS and DFS in HCC patients.

The maker of proliferation Ki-67 expresses in all phases of the cellular cycle over than G_0_ phase.^[63]^ MKI67 protein expression in carcinomas has been intensively investigated, and the MKI67-positive cell rate has been shown to be associated with clinical-pathological features and even clinical outcomes in various cancers, including HCC.^[64]^ In a study of patients undergoing surgical resection for HCC, higher levels of MKI67 expression in tumor tissue were associated with a higher tumor grade and early tumor recurrence.^[65]^ Furthermore, staining for MKI67 and P53 are widely used to predict the clinical outcomes of HCC patients after resection and liver transplantation.^[66]^

EZH2 is a member of the polycomb group (PcG) protein family, which modifies transcription at the epigenetic level by regulating histone and DNA methylation.^[67,68]^ Lots of studies have shown that many tumor suppressor genes are suppressed by EZH2 in malignancies and that EZH2 dysregulation plays a crucial role in carcinogenesis.^[69,70]^ In our study, the expression of EZH2 was higher in HCC tumor tissue, and the high expression of EZH2 was associated with unfavorable OS and DFS in HCC patients.

CDC6 plays a critical role in the initiation of DNA replication. As cells enter the G1 phase, CDC6 binds to the origin recognition complex and initiates the assembly of the pre-replicative complex (pre-RC) with chromatin licensing and DNA replication factor 1 and mini-chromosome maintenance proteins.^[71,72]^ Once phosphorylated by CDKs at the G1/S phase, CDC6 is released from the pre-RC and then DNA is licensed for replication. Growing evidence have suggested that deregulation of CDC6 may contribute to cancer initiation and progression.^[73]^ Overexpression of the CDC6 protein has been observed in different types of cancer.^[74]^ Our study reveal that the expression of CDC6 was higher in HCC tumor tissue and the high expression of CDC6 was related to unfavorable OS and DFS in HCC patients.

TOP2A, is a key nuclease that facilitates the temporary cleavage and ligation cycle of DNA.^[75]^ In all forms of topoisomerases, TOP2A is predominantly involved in proliferating cells and overexpressed in a variety of cancers (such as breast cancer, urinary bladder cancer, and ovarian carcinoma).^[75]^ For HCC, bioinformatics analysis showed that overexpression of TOP2A was common in HCC tumor tissues relative to those in normal liver tissues.^[76]^ Moreover, Wong et al found that the high expression of TOP2A was correlated with microvascular invasion, advance histological grading, chemotherapy resistance, and poor survival rate.^[77]^ In our study, the expression of TOP2A was higher in HCC tumor tissue compared to normal liver tissue, and associated with unfavorable OS and DFS in HCC patients.

A list of 29 drugs with potential therapeutic efficacy against HCC was identified through the DGIdb database. Among the 10 hub genes, the potential gene targeting the drugs are AURKB, EZH2, and TOP2A. In Table 3, most of the drugs were inhibitors of AURKB, EZH2, and TOP2A. Some researchers also have identified similar molecules, such as phenoxybenzamine, emetine, and fendiline, which may be effective drugs against HCC.^[78]^ Meanwhile, there are some existing clinical trials based on these molecules.^[79,80]^ However, only a few of them have been used for HCC. More studies and clinical trials were needed to identify and explore the effective drugs for HCC. Nevertheless, the present study might push new valuable insights into the individualized and targeted therapy for HCC, and the identified conventional drugs were of potential new use.

## 5 Conclusions

In summary, the study identified commonly changed 197 DEGs in HCC through using integrated bioinformatics analysis, including 54 upregulated DEGs and 143 downregulated DEGs. And 10 hub genes(FOXM1, AURKA, CCNA2, CDKN3, MKI67, EZH2, CDC6, CDK1, CCNB1, and TOP2A) might play important roles in HCC. The expression of the hub genes was revealed to be increased in HCC, and the overexpression level predicted a poor prognosis. The 10 hub genes might function as novel markers and/or targets for the early HCC detection, prognostic judgment, and targeted therapy of HCC. Additionally, a number of drugs targeting the hub genes were identified, and they could be potentially utilized for the treatment of HCC patients. This study provided a powerful basis for HCC studies, and further experimental studies were needed.

## Acknowledgments

We sincerely thank the GEO, Enrichr, STRING, GEPIA, TCGA, HAP, cBioPortal, Kaplan–Meier plotter, DGIdb, and STITCH databases for providing their platforms and contributors for their valuable data.

## Author contributions

Concept and design: Ping Huang; analysis and interpretation of the data: Xiaolong Chen; acquisition of data: Xiaolong Chen and Zhixiong Xia; making diagrams and tables of the article: Xiaolong Chen and Yafeng Wan; drafting of the article: Xiaolong Chen and Zhixiong Xia; critical revision and final approval of the article: Ping Huang.

**Conceptualization:** Ping Huang.

**Data curation:** Xiaolong Chen.

**Formal analysis:** Xiaolong Chen.

**Funding acquisition:** Ping Huang.

**Investigation:** Xiaolong Chen.

**Methodology:** Xiaolong Chen, Yafeng Wan.

**Resources:** Zhixiong Xia.

**Software:** Zhixiong Xia.

**Supervision:** Ping Huang.

**Validation:** Ping Huang.

**Visualization:** Xiaolong Chen, Zhixiong Xia, Yafeng Wan.

**Writing – original draft:** Xiaolong Chen.

**Writing – review & editing:** Ping Huang.

## Supplementary Material

Supplemental Digital Content

## Supplementary Material

Supplemental Digital Content

## Supplementary Material

Supplemental Digital Content

## Supplementary Material

Supplemental Digital Content

## Supplementary Material

Supplemental Digital Content

## Supplementary Material

Supplemental Digital Content
