# Supplementary material for: Identification of hub genes and candidate drugs in hepatocellular carcinoma by integrated bioinformatics analysis
Source: Medicine (Baltimore). 2021 Oct 1;100(39):e27117. doi: 10.1097/MD.0000000000027117 (PMC8483840; doi:10.1097/MD.0000000000027117)

**Fig. S1** PPI network construction. PPI network was constructed by all the 197 DEGs using STRING database. Nodes represent the productions of DEGs. Edges represent the protein-protein associations. Bluish edges represent that the associations are from curated databases, purple edges represent the associations are experimentally determined, green edges represent gene neighborhood. Red edges represent gene fusions, blue edges represent gene co-occurrence, yellow edges represent textmining, black edges represent co-expression. DEGs, differentially expressed genes.


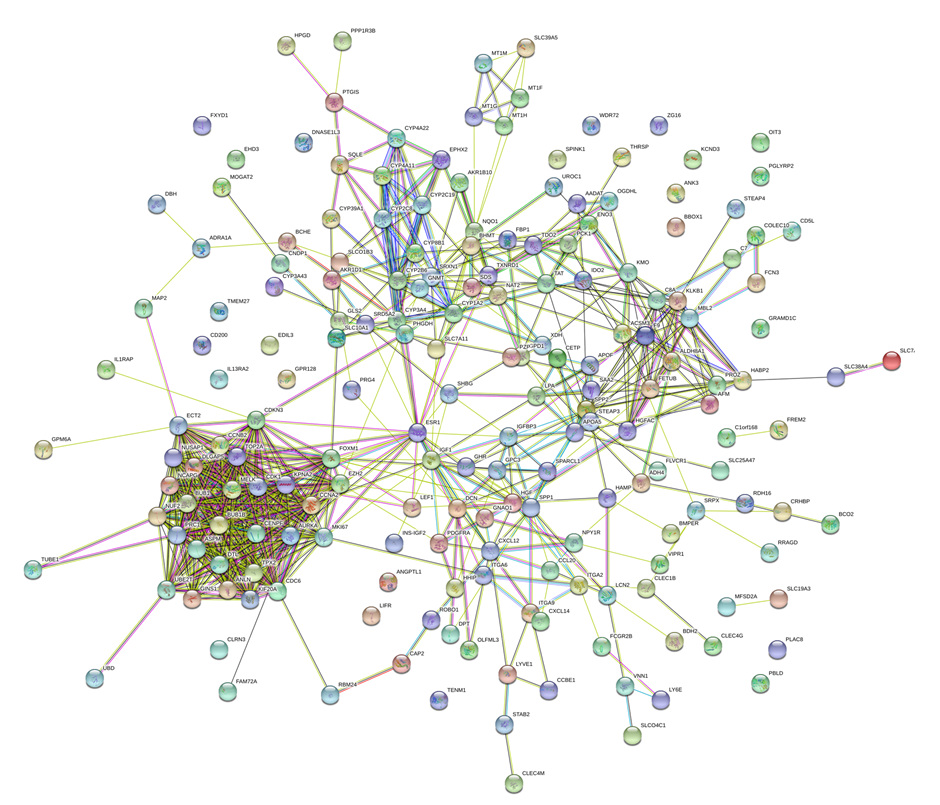

Supplement: Supplemental Digital Content [file medi-100-e27117-s001.doc]
