# Supplementary material for: Identification of hub genes and candidate drugs in hepatocellular carcinoma by integrated bioinformatics analysis
Source: Medicine (Baltimore). 2021 Oct 1;100(39):e27117. doi: 10.1097/MD.0000000000027117 (PMC8483840; doi:10.1097/MD.0000000000027117)

**Fig. S2** Representative immunohistochemistry images of (A) FOXM1, (B) AURKA, (C) CCNA2, (D) MKI67, (E) EZH2, (F) CDC6, (G) CDK1, (H) CCNB1, and (I) TOP2A in HCC and non-cancerous liver tissues derived from the HPA database. HCC, hepatocellular carcinoma; HPA, Human Protein Atlas.


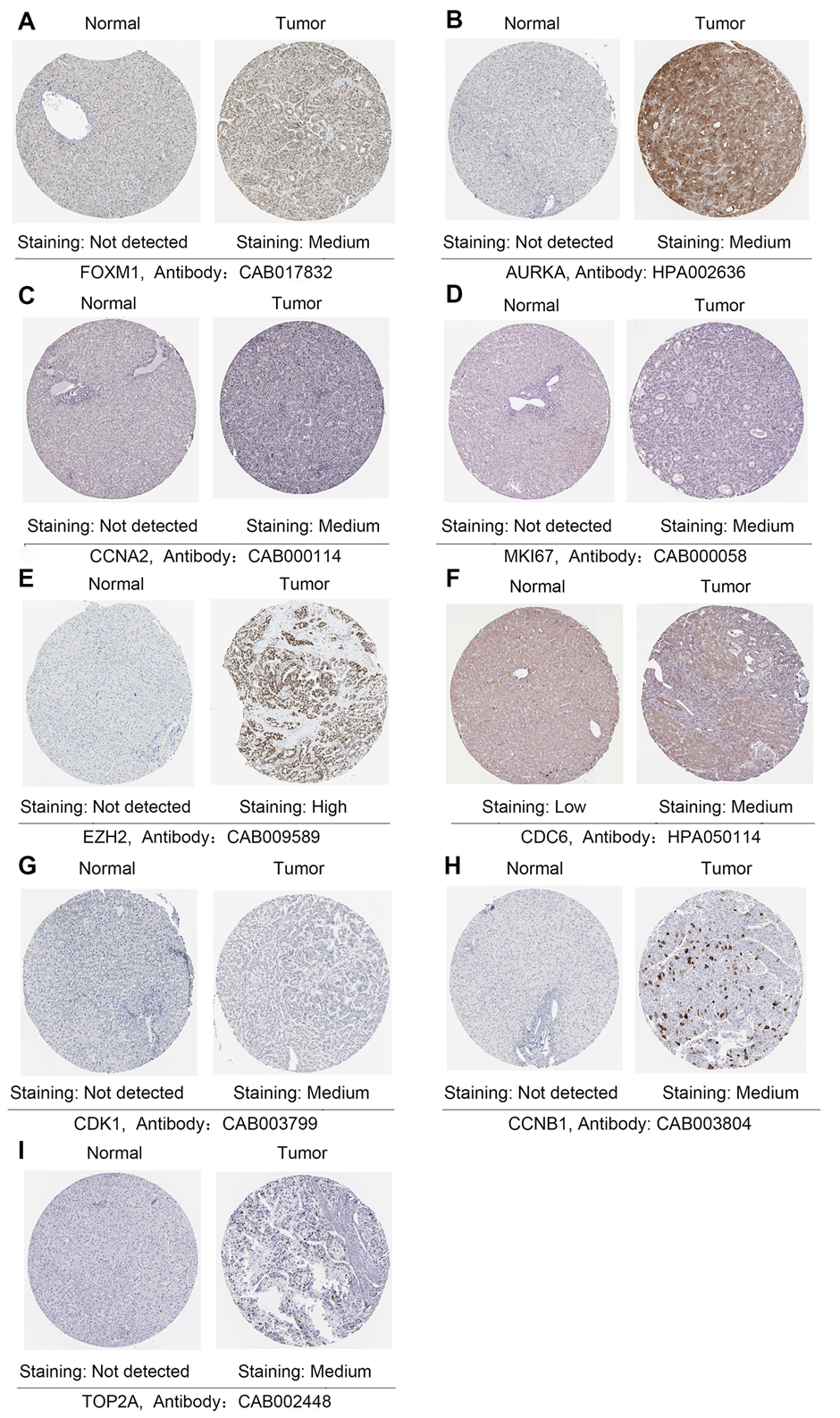

Supplement: Supplemental Digital Content [file medi-100-e27117-s002.doc]
