# Supplementary material for: Identification of hub genes and candidate drugs in hepatocellular carcinoma by integrated bioinformatics analysis
Source: Medicine (Baltimore). 2021 Oct 1;100(39):e27117. doi: 10.1097/MD.0000000000027117 (PMC8483840; doi:10.1097/MD.0000000000027117)

**Fig. S3** DFS of LIHC patients overexpressed the 10 hub genes were analyzed by the GEPIA online database. Data are presented as log-rank P and the hazard ratio with a 95% confidence interval. FOXM1, log-rank P=0.00066; AURKA, log-rank P=0.0012; CCNA2, log-rank P=0.0037; CDKN3, log-rank P=0.0074; MKI67, log-rank P=4.2e-05; EZH2, log-rank P=1e-04; CDC6, log-rank P=0.0044; CDK1, log-rank P=0.00057; CCNB1, log-rank P=2.8E-06; and TOP2A, log-rank P=0.00053. Log-rank P<0.01 was considered statistically significant. DFS, disease-free survival; LIHC, liver hepatocellular carcinoma.


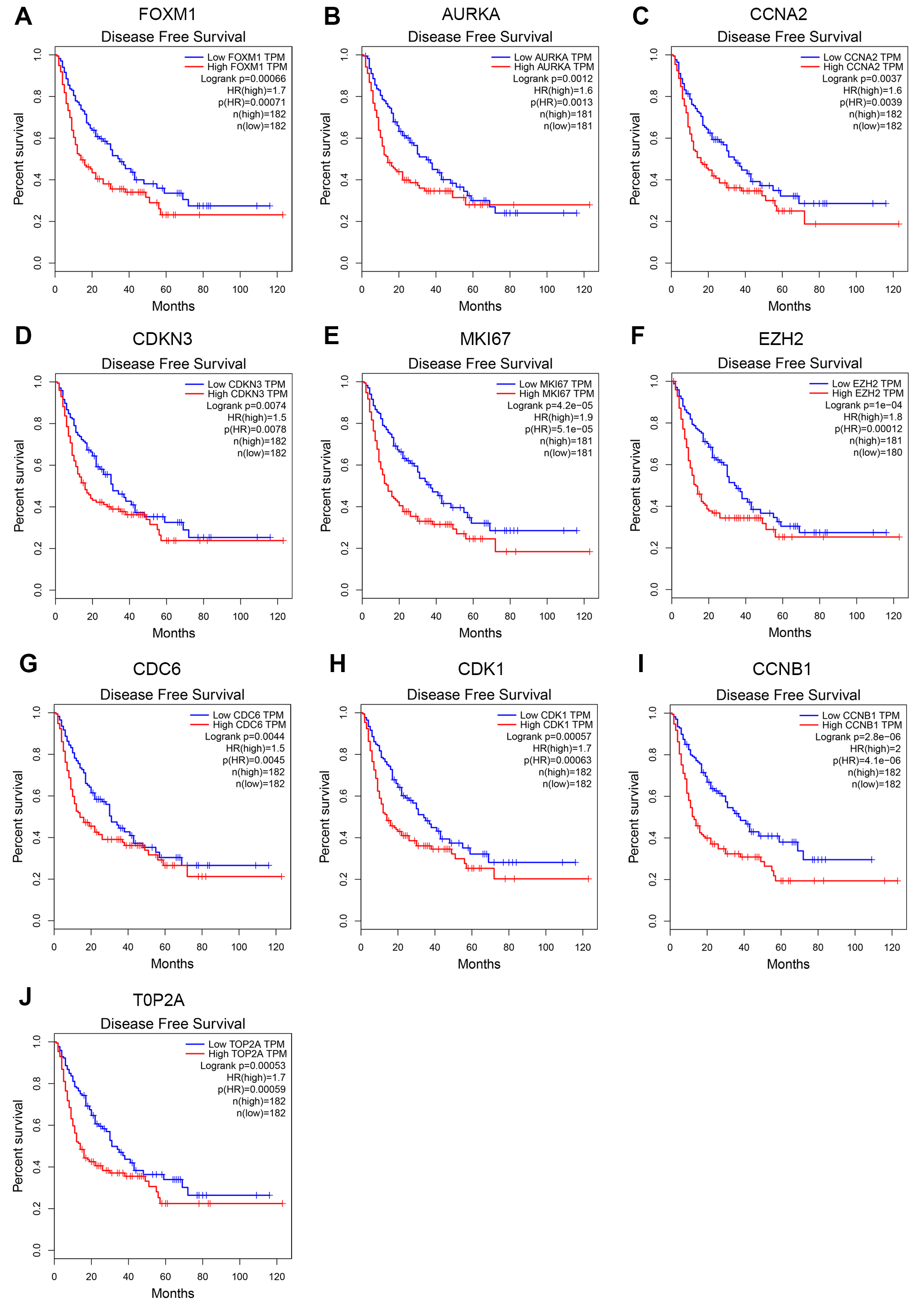

Supplement: Supplemental Digital Content [file medi-100-e27117-s003.doc]
