# Supplementary material for: Identification of hub genes and candidate drugs in hepatocellular carcinoma by integrated bioinformatics analysis
Source: Medicine (Baltimore). 2021 Oct 1;100(39):e27117. doi: 10.1097/MD.0000000000027117 (PMC8483840; doi:10.1097/MD.0000000000027117)

**Fig. S4** Drug-hub genes network of AURKA. Inhibition of URKA may have influence on TPX2, microtubule nucleation factor (TPX2), cell division cycle 20 (CDC20), tumor protein p53 (TP53), cell division cycle 25B (CDC25B), baculoviral IAP repeat containing 5 (BIRC5).


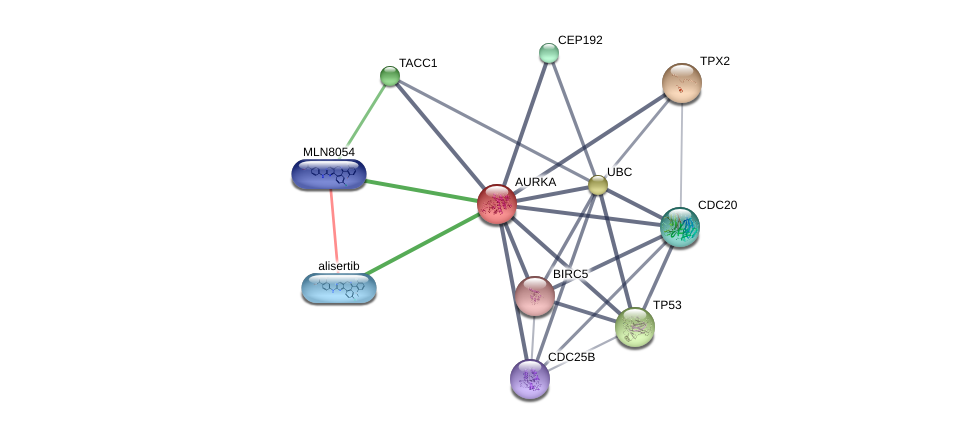

Supplement: Supplemental Digital Content [file medi-100-e27117-s004.doc]
