# Supplementary material for: Identification of hub genes and candidate drugs in hepatocellular carcinoma by integrated bioinformatics analysis
Source: Medicine (Baltimore). 2021 Oct 1;100(39):e27117. doi: 10.1097/MD.0000000000027117 (PMC8483840; doi:10.1097/MD.0000000000027117)

**Fig. S5** Drug-hub genes network of EZH2. Inhibition of EZH2 may have influence on histone deacetylase 1 (HDAC1), BMI1 proto-oncogene, polycomb ring finger (BMI1), YY1 transcription factor (YY1), DNA methyltransferase 3 alpha (DNMT3A), DNA methyltransferase 3 beta (DNMT3B), DNA methyltransferase 1(DNMT1), RB binding protein 4(RBBP4), embryonic ectoderm development(EED).


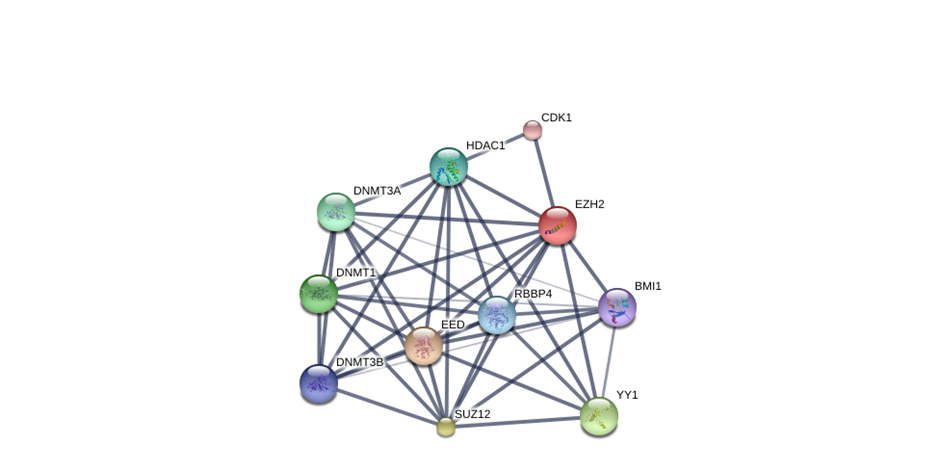

Supplement: Supplemental Digital Content [file medi-100-e27117-s005.doc]
