# Supplementary material for: Identification of hub genes and candidate drugs in hepatocellular carcinoma by integrated bioinformatics analysis
Source: Medicine (Baltimore). 2021 Oct 1;100(39):e27117. doi: 10.1097/MD.0000000000027117 (PMC8483840; doi:10.1097/MD.0000000000027117)

**Fig. S6**  Drug-hub genes network of TOP2A. Inhibition of TOP2A may have influence on DNA topoisomerase I (TOP1), DNA topoisomerase II beta (TOP2B), ubiquitin C (UBC), proliferating cell nuclear antigen (PCNA), small ubiquitin-like modifer 1 (SUMO1), and SUMO2.


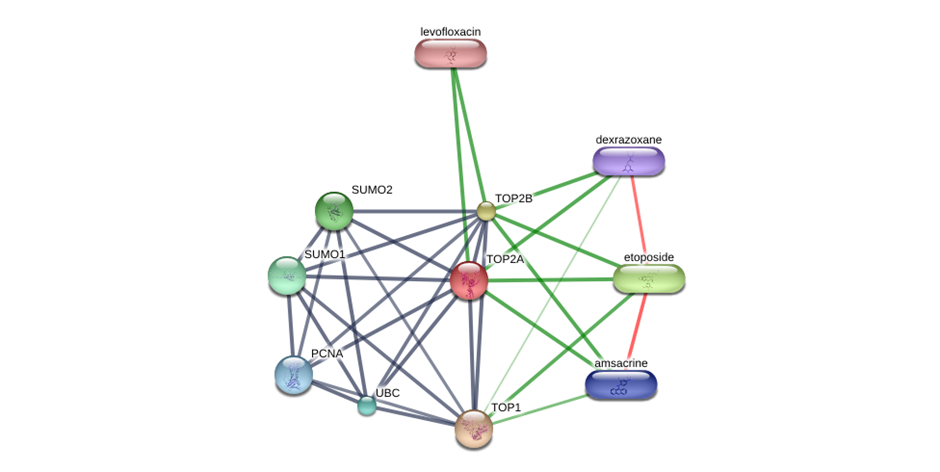

Supplement: Supplemental Digital Content [file medi-100-e27117-s006.doc]
